# Determination of Mycotoxins in Dried Fruits Using LC-MS/MS—A Sample Homogeneity, Troubleshooting and Confirmation of Identity Study

**DOI:** 10.3390/foods11060894

**Published:** 2022-03-21

**Authors:** Kai Zhang, Steven Tan, David Xu

**Affiliations:** 1 US Food and Drug Administration, Center for Food Safety and Applied Nutrition, Office of Regulatory Science, HFS-717. 5001 Campus Drive, College Park, MD 20740, USA; steven.tan@fda.hhs.gov; 2 Joint Institute for Food Safety and Applied Nutrition, University of Maryland, 2134 Patapsco Building, 5145 Campus Drive, College Park, MD 20740, USA; david.xu@fda.hhs.gov

**Keywords:** mycotoxins, dried fruits, LC-MS

## Abstract

To monitor co-exposure to toxic mycotoxins in dried fruits, it is advantageous to simultaneously determine multiple mycotoxins using a single extraction and liquid chromatography with tandem mass spectrometry (LC-MS/MS) analysis. In this study, we applied a stable isotope dilution and LC-MS/MS method to multi-mycotoxin analysis in dried fruits, selecting raisins, plums, figs, and cranberries for matrix extension. Samples were prepared using cryogenic grinding, followed by the fortification of carbon-13 (^13^C) uniformly labeled internal standards for twelve mycotoxins, and extraction using 50% acetonitrile. Homogeneity of prepared samples, defined as particle size Dv90 < 850 µm for the tested matrices, was characterized using a laser diffraction particle size analyzer, and reached using cryogenic grinding procedures. The majority of recoveries in the four matrices for aflatoxins and ochratoxin A spiked at 1–100 ng/g; fumonisins, T-2 toxin, HT-2 toxin, and zearalenone spiked at 10–1000 ng/g, ranged from 80 to 120% with relative standard deviations (RSDs) of <20%. Deoxynivalenol was not detected at 10 and 100 ng/g in plums, and additional troubleshooting procedures using liquid-liquid extraction (LLE), solid phase extraction (SPE), and elution gradient were evaluated to improve the detectability of the mycotoxin. Furthermore, we confirmed the identity of detected mycotoxins, ochratoxin A and deoxynivalenol, in incurred samples using enhanced product ion scans and spectral library matching.

## 1. Introduction

Dried fruits such as raisins, prunes, figs, and cranberries are produced by sun drying or other dehydration procedures and are well-known for their sweet taste and longevity of storage [1,2]. As a popular food in the U.S. and in European Union (EU) countries, dried fruits are often consumed as snacks and used in recipes for yogurt, trail mix, salads, cereals, and baked goods to enhance their taste. The annual consumption of raisins in the EU and the US is approximately 330,000 tons and 220,000 tons, respectively. To meet consumers’ needs, more than 260,000 tons of raisins and 54,000 tons of prunes are produced annually in the US, and 335,000 tons of raisins are imported by EU countries [3].

Dried fruits are susceptible to mold development and mycotoxin contamination, depending on processing and storage conditions. Consumption of dried fruits contaminated by mycotoxins can lead to adverse health effects [4,5,6]. Since complete removal of mycotoxins through current production and processing methods for dried fruits is not feasible [7], the U.S. Food and Drug Administration (FDA) and the EU have established regulatory levels for toxic mycotoxins in foods. To comply with established regulations in support of consumer safety, the analysis of mycotoxins has become an essential part of regulatory monitoring and surveillance programs [8,9]. According to Rapid Alert System for Food and Feed (RASSF) annual reports from 2016–2020, more than 50 notifications were issued annually due to violative findings of aflatoxins and/or ochratoxin A in dried fruits imported by EU countries [10,11,12,13,14].

Over the years, many methods have been developed for the determination of mycotoxins in dried fruits. Due to the diverse physicochemical properties of mycotoxins, the extraction, clean-up, chromatographic separation, and detection procedures of conventional enzyme-linked immunosorbent assay (ELISA), thin layer chromatography (TLC), or liquid chromatography coupled to ultraviolet or fluorescence detector (LC-UV/FLD) methods were only suited for the analysis of a single mycotoxin or single class of mycotoxins [4,15]. As different mycotoxins are often present in the same dried fruit product, analytical methods that can simultaneously determine different mycotoxins are seen as more efficient. With advances in LC-MS instrumentation, LC-MS-based, especially liquid chromatography with tandem mass spectrometry (LC-MS/MS)-based, analysis has received increasing attention since the early 2000s. Compared to ELISA, TLC, or LC-UV/FLD, LC-MS/MS methods not only offer superior sensitivity and specificity, but also can simultaneously determine multiple mycotoxins in a single analysis [16,17,18].

Coupled with stable isotope dilution assay, LC-MS/MS methods can efficiently address one challenging issue related to LC-MS-based mycotoxin analysis, matrix-induced signal effects, eliminating the preparation of matrix-matched calibration standards [19,20,21]. Furthermore, if isotope-labeled internal standards are spiked into samples prior to extraction, the approach could offset insufficient extraction efficiency when using one generic extraction solvent to target multiple mycotoxins [22]. In our previous studies, we demonstrated the applicability of stable isotope dilution LC-MS/MS in cereal grains, animal feeds, and fatty food matrices. To further harness the benefits of stable isotope dilution LC-MS/MS, we decided to extend its applications to dried fruits in this study.

Mycotoxin analysis consists of sampling plans (e.g., sample collection), sample preparation (e.g., comminution-particle size deduction), subsampling for test portion, and instrumental analysis (e.g., identification and quantitation). During sample preparation and subsampling, it is imperative to characterize sample homogeneity, minimizing the impact of compositional heterogeneity (“hot spot”) of mycotoxins on qualitative and quantitative results; however, homogeneity tests and data are often excluded from method development studies due to the lack of practical protocols or tools. Compared to cereal grains, it is more challenging to produce homogeneous composites for dried fruits so in this study, we developed a cryogenic milling procedure to achieve homogeneity and characterized the homogeneity of prepared dried fruit samples based on particle size distribution. Particle size analysis was conducted using a laser diffraction particle size analyzer. Compared to existing sieving or ISO protocols [23], laser diffraction technology provides a more time-efficient and less labor-intensive approach to characterizing particle size. We therefore found its applicability to be worth exploring in our study.

## 2. Materials and Methods

### 2.1. Chemicals

LC grade acetonitrile, methanol, and water were purchased from Thermo Fisher Scientific (Waltham, MA, USA). Mycotoxin stock solutions I, II, and III were obtained from Romer Labs (Union, MO, USA). Solution I contained 10 μg/mL each of aflatoxin B_1_ (AFB1), aflatoxin B_2_ (AFB2), aflatoxin G_1_ (AFG1), aflatoxin G_2_ (AFG2), and ochratoxin A (OTA) in methanol; Solution II contained 100 μg/mL each of fumonisin B_1_ (FB1), fumonisin B_2_ (FB2), and fumonisin B_3_ (FB3) in acetonitrile–water (1 + 1, *v/v*); Solution III contained 100 μg/mL each of deoxynivalenol (DON), HT-2 toxin (HT-2), T-2 toxin (T-2), and zearalenone (ZON) in acetonitrile. The following carbon-13 (^13^C)-IS stock solutions were purchased from Romer Labs: ^13^C_17_-AFB1 + B2 + G1 + G2 (0.5 μg/mL), ^13^C_20_-OTA (10 μg/mL), ^13^C_34_-FB1 (25 μg/mL), ^13^C_34_-FB2 (10 μg/mL), ^13^C_34_-FB3 (10 μg/mL), ^13^C_15_-DON (25 μg/mL), ^13^C_22_-HT-2 (25 μg/mL), ^13^C_24_-T-2 (25 μg/mL), and ^13^C_18_-ZON (25 μg/mL).Spiking solutions of ^13^C-IS and calibration standards were prepared according to a previously described protocol [22].

### 2.2. Cryogenic Milling

Samples were stored at −80 °C overnight. On the next day, coarse samples (approximately 500 g) were prepared by mixing the dried fruits with dry ice and blended in a Blixer 4 blender (Robot Coupe, Inc., Ridgeland, MS, USA) until a powdery consistency was achieved. Prepared coarse samples were transferred into polypropylene bags and stored at −20 °C in dark conditions. The final homogenized sample was prepared by mixing coarse samples (25 g each) with approximately 25 g dry ice and homogenized using an IKA tube mill (IKA Works, Inc., Wilmington, NC, USA) at 25,000 rpm for 1.5 min. After carbon dioxide was sublimed, the homogenized samples were used for recovery and homogeneity tests.

### 2.3. Sample Preparation

Fortified dried fruit samples were prepared following fortification of ^13^C-IS, extraction, centrifugation, and filtration [22]. Briefly, the target mycotoxins were spiked into figs, cranberries, plums, and raisins (1.00 ± 0.05 g each), achieving the final concentrations of AFB1, AFB2, AFG1, AFG2, and OTA at 1, 10, and 100 ng/g and FBs, DON, HT-2, T-2 and ZON at 10, 100, and 1000 ng/g. Three replicates were prepared for each concentration, including matrix blanks. Prior to extraction, each sample was fortified with the ^13^C-IS spiking solution (100 μL). Then samples were extracted, centrifuged, and filtered, followed by LC-MS analysis.

### 2.4. Particle Size Analysis

The particle sizes of dried fruit samples after cryogenic milling were measured using a Malvern Mastersizer 3000 (Malvern Panalytical Inc. Westborough, MA, USA) equipped with two laser sources, 470 nm and 633 nm. As dried fruit samples cannot be dispersed by air, we developed a wet measurement procedure, with methanol used as the dispersant. Samples (approximately 0.5 g each) were gradually introduced into the particle size analyzer using a Hydro LV Disperser (Malvern Panalytical Inc.). Samples were dispersed for 30 s before 10 individual measurements were recorded at an obscuration reading between 10% and 30%. The refractive index was set at 1.6. To suspend the sample in methanol, the stirring rate of the disperser was set at 3500 rpm. Particle size distribution was reported as the mean and relative standard deviation of the volume densities, Dv10, Dv50, and Dv90 (particles account for 10, 50 and 90% of the total sample volume) for each measurement. Reference material CRM 3310 (Malvern Panalytical Inc.) and SRM 1565 (National Institute of Standards and Technology; NIST) were analyzed along with the dried samples for quality control (QC).

### 2.5. Troubleshooting Procedures for the Determination of DON in Plums

To improve the detectability of the selected mycotoxin, we conducted and compared three troubleshooting procedures. Sample extracts of matrix blanks and spiking replicates (*n* = 3) at 100 and 1000 ng/g in plum were prepared following the dilution, extraction and filtration procedures described in the Sample preparation section above.

- Liquid-liquid extraction (LLE): Sample extracts (0.5 mL, 50% ACN) were mixed with dichloromethane (DCM) (1.0 mL) in a 15 mL tube, which was vigorously vortexed for 1 min and left at room temperature for 2 min until phase separation was observed. Half a milliliter of the bottom layer (DCM phase) was pipetted into a 1.5 mL LC vial for LC-MS analysis.
- Solid phase extraction (SPE): SPE was conducted using a vacuum manifold with a collection rack inserted inside. Florisil SPE cartridges (1.0 mL, 100 mg, Restek, College Station, PA, USA) were conditioned sequentially with 0.5 mL of isopropyl alcohol (IPA)/DCM (*v/v*, 50/50) and hexane. After an aliquot (0.5 mL) of the sample extracts was loaded, the SPE cartridges were washed using 5% DCM in hexane (0.5 mL) and DON was eluted and collected using 1.0 mL DCM in the 15 mL disposable centrifuge tubes on the collection rack. Elution rate was controlled at approximately 2 drops/s.
- LC separation: The elution gradient of the original LC method was evaluated to improve the separation of target analytes from matrix interreferences. The column, mobile phases, flow rate, injection volume, and column temperature of the method remained the same. In the original LC method, 10 mM ammonium formate/0.1% formic acid/water (A) and 10 mM ammonium formate/0.1% formic acid/methanol (B) were used as mobile phases. Gradient elution started at 5% B, ramped to 40% B in 2 min and then to 100% B by 10 min via linear gradient mode, held for 2.5 min, and changed to 5% B at 12 min, followed by 3 min of column conditioning. The modified gradient elution started at 5% B and held for 4 min, ramped to 100% B by 12 min via linear gradient mode, held for 2.5 min, and changed to 5% B at 15 min, followed by 3 min of column conditioning. Same flow rate, 0.3 mL/min was used for both gradients.

### 2.6. LC-MS Information Dependent Analysis (IDA) and Enhanced Product Ion (EPI) Analysis

We performed the analysis following a previously reported protocol [24]. Identical LC conditions were used for the LC-MS-IDA-EPI and LC-MS/MS analyses. Sciex OS, LibraryView, and Mycotoxin Library 1.0 (SCIEX, Framingham, MA, USA) were used as the source libraries to match EPI spectra collected from incurred samples, which were collected at a collision energy of 45 eV with a collision energy spread (CES) of 30 eV (45 ± 30 eV). Collision gas (N_2_) pressure was set to “high.” EPI spectra were collected within a range from *m/z* = 50 to the precursor *m/z* plus 50 amu. The fill time of the ion trap was determined using dynamic fill time function. Product ions were scanned out of the 6500 QTrap at a rate of 10,000 amu/s. Source temperatures and voltages were the same as those used for the LC-MS/MS analysis. For the library searches, the mass tolerance window for precursor and fragment ions was set at ±0.4 amu. The retention time window was ±45 s.

## 3. Results and Discussion

### 3.1. Homogeneity Test

In general, a test portion used for LC-MS analysis only accounts for a small portion of a sample. To ensure the test portion reflects the occurrence and concentrations of target mycotoxin in the sample, great care must be taken to first homogenize the sample, then test it for homogeneity using ISO protocols [23] or sieving. These approaches, however, may be cumbersome for routine sample analysis, so it is necessary to explore and adopt alternative technologies to characterize the homogeneity of processed samples in an efficient and practical manner. Laser diffraction technology can measure particles ranging in size from nanometers to millimeters. Well-established mathematical models and protocols can estimate particle size distribution based on measured scattering light intensity [25]. Owing to its repeatability, ease of verification, and speed of measurement, the technology has been widely used by the pharmaceutical, mining, and cosmetic industries [26,27]. Therefore, in this study we used a laser diffraction particle size analyzer to characterize homogeneity of dried fruits after cryogenic milling.

Table 1 summarizes particle size distribution of cranberries, figs, plums, and raisins prepared using cryogenic milling. Average particle sizes and RSDs (*n* = 10) are reported as three individual percentiles of cumulative sample volume distribution: Dv10, Dv50, and Dv90. NIST 1565 (corn) and CRM 3310 (glass beads) were used as QC to ensure the performance of the wet measurement procedure and the instrument. The largest particle sizes (Dv90) ranged from 427 µm (raisin) to 567 µm (fig), which is below the threshold of #20 sieve (850 µm) recommended by AOAC International [28]. Except for the RSD of raisin samples at Dv90, the other RSDs were below 10%, suggesting satisfactory repeatability. The measured value of NIST 1565, 550 µm with RSD < 5%, is in good agreement with the reference value, 588 µm. Relative differences between the measured values and the reference values of CRM 3310 are within 10%.

**Figure 1:**
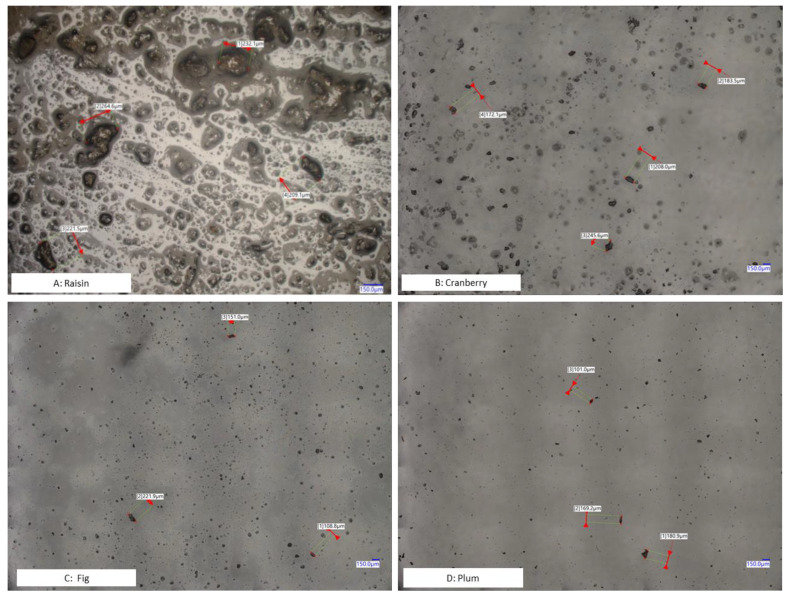
Microscopic images (Digital Microscope, Keyence VHX-7000 Series) of dispersed dried fruit samples prepared using cryo-milling.

In addition, we also conducted microscopic inspections to check sample particle sizes after cryogenic milling. Cryo-milled cranberry, fig, plum, and raisin samples were applied on microscope slides for visual inspection. The micrographs confirmed the findings of the particle size distribution (Figure 1). These results suggest that the current cryogenic milling process can reach sample homogeneity, defined as particle size < 850 µm.

Continuous improvement in the sensitivity of LC-MS instruments eliminates the need to extract and concentrate a larger test portion, e.g., 50 or 100 g. For LC-MS based mycotoxin determination, test portions range from 0.25–5 g [29,30,31,32]. The use of a smaller test portion leads to simpler sample preparation (e.g., dilute-and-shoot) and significant reduction in organic solvents but may also require a higher degree of homogeneity (smaller particle size). According to Gy’s sampling theory [33], to maintain a defined standard sampling error (fundamental error), homogeneity of the sample dictates the minimum test portion that should be used, and the latter is closely correlated with the particle size of the sample. Whether a universal homogeneity threshold, (e.g., particle size < 850 µm), can be applied to various test portions used for multi-mycotoxin analysis in different food matrices is an ongoing debate, as the combinations of individual mycotoxins and matrices may show different levels of homogeneity (Figure 1 and Table 1). Although the effort required to define practical mycotoxin/matrix-dependent homogeneity criteria may be overwhelming and is beyond the scope of this study, we believe it is necessary to systematically evaluate the impact of homogeneity on each mycotoxin/matrix pair in the future.

### 3.2. Recovery Studies

The majority of the spike recoveries of the 12 targeted mycotoxins spiked in cranberry, fig, plum, and raisin ranged from 80–120%, with RSDs < 20% (Table 2). In general, the results are consistent with previous spike recoveries in corn, wheat flour and peanut butter [22]. High spike recovery of fumonisin B1 at 10 ppb in plum was due to the content of approximately 10 ppb fumonisin B1 in samples used for recovery studies. After subtraction of FB1 in the sample, the spike recovery at 10 ppb would be around 155%. HT-2 (10 ppb, cranberry) and FB3 (10 ppb, fig) were defined as “not detected (ND)” because their relative ion ratios were out of the allowable range required by FDA identification criteria [34]. DON was not detected in plum spiked at 10 or 100 ppb, though 13C-DON (spiked at 100 ppb) was detected. Co-extracted/eluted matrix interferences suppressed the signal of DON. The advisory level of DON established by the FDA is 1.0 ppm [35] and the Codex Alimentarius Commission adopted a maximum level of 0.2 ppm for DON in cereal-based foods for infants and young children [36]. Without any further clean-up, the estimated LOQ for DON is above 100 ppb. To ensure the method can quantitate DON below 200 ppb in dried plums, we performed additional clean-up or modification of chromatographic separation to achieve better sensitivity for this regulated mycotoxin.

### 3.3. Determination of DON Using Liquid-Liquid Extraction and LC Separation

**Figure 2:**
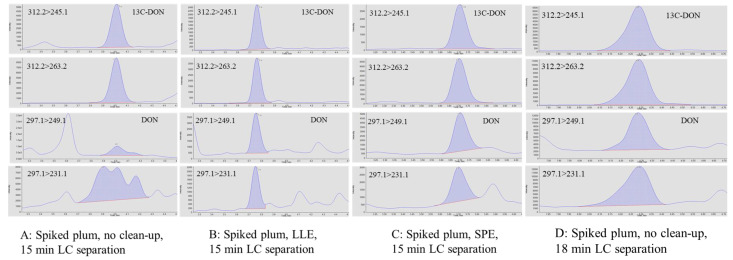
Detection of 13C-DON and DON spiked in plum using different clean-up and LC separation conditions. (**A**): without additional clean-up, no detection of DON with the 15 min LC method. (**B**): sample prepared using LLE, DON detected; no modification of the 15 min LC method. (**C**): sample prepared using LLE, DON detected; no modification of the 15 min LC method. (**D**): without any clean-up, DON detected with an 18 min LC method.

In multiple reaction monitoring (MRM) methods, two structurally specific transitions are generally selected for the identification of each analyte, in the hope that coupled with LC, the selection could provide sufficient specificity. Unfortunately, without any prior knowledge of sample matrices, the two pre-selected MRM transitions cannot always guarantee that the detection of target analyte would be free from interferences. In the presence of isobaric interferences that could not be separated from DON on the LC column, the detection of DON at 10 and 100 ppb in plums was compromised. On the contrary, without interferences, 13C-DON at 100 ppb was clearly detected in the same extract (Figure 2A).

The removal of interfering matrix components can be achieved using sample clean-up techniques (SPE, LLE, immunoaffinity columns), different chromatographic conditions (columns or gradient conditions), and/or modifications of MS/MS parameters. Using a simple extraction without any in-depth clean-up procedures, DON is susceptible to interferences caused by co-extracted matrix components. To remedy this approach in select food matrices, our first approach focused on sample clean-up.

We performed LLE on the plum extracts (50% acetonitrile) using dichloromethane (DCM), a polar organic solvent that is immiscible in water [37]. Using a LLE, DON is extracted into the DCM phase, separating from more water soluble matrix interferences. In contrast with conventional LLE, no separatory funnel was used in this study. The extraction was conducted in a 15 mL tube using a small amount of DCM (1.0 mL) and sample extract (0.5 mL). Phase separation was easily formed. The analysis of the DCM extracts clearly showed the detection of DON without any interferences (Figure 2B). Since 13C-DON was used as the internal standard and was co-extracted with DON, volume change in the DCM phase did not affect quantitation of DON. The corresponding spike recoveries of DON, spiked at 100 and 1000 ng/g, were 106% and 103%, respectively, with RSDs ≤ 5% (*n* = 3). Alternatively, SPE (Figure 2C) could be used to achieve the purification of DON from the plum extracts. Spike recoveries at 100 and 1000 ng/g were 97% (RSD = 3%, *n* = 3) and 109% (RSD = 6%, *n* = 3), respectively, but the approach requires conditioning, washing, and elution steps, leading to a more time-consuming procedure without any additional benefits to the LLE approach.

The third approach focused on LC conditions. Based on the LLE results, the interferences were more polar than those of DON and were more likely to partition into the aqueous phase. Therefore, to separate DON and the interferences, a 4 min isocratic elution using 5% methanol (*v/v*, methanol/water) was added to the beginning of the original gradient program, in which the mobile phase was directly ramped from 5% to 40% methanol within the first 2 min. The modification slowed the change in the polarity of the mobile phase, resulting in a longer retention time of DON. With different retention properties, DON was detected due to separation of DON and the interferences (Figure 2D). The corresponding spike recoveries of DON spiked at 100 and 1000 ng/g were 83% and 91%, respectively, with RSDs < 5% (*n* = 3).

LC-MS based analysis of mycotoxins has improved in scope and sensitivity due to advancements in new instrument technologies, in parallel with generic extraction methods. However, without optimizing conditions for each mycotoxin, one cannot always expect satisfactory results for all targeted mycotoxins in all matrices. Troubleshooting may be required for individual mycotoxins for extension of existing methods to additional matrices. The above two approaches demonstrated that after practical modifications of sample preparation or LC conditions, DON was quantitatively recovered at desired concentration levels with marginal additional effort. Finding a reasonable balance between time consuming troubleshooting efforts and data quality needs depends not only on method performance, but also method validation/modification criteria [38].

### 3.4. Analysis of Incurred Samples and Confirmation Using LC-MS-EPI Library

We analyzed twenty market samples (three cranberry, three fig, three raisin, three plum, three date, two fruit mix, one cherry, one kiwi, one peach products) purchased from local and online stores. OTA (3.5 ± 0.5 ng/g, *n* = 3) was detected in a fig sample and DON (119 ± 3 ng/g, *n* = 3) was detected in a dried fruit mix. No mycotoxins were detected in the other samples. This study encompassed only a very small convenience sampling, and the reported results should not be interpreted in any way as representative of the entire market.

Identification of mycotoxins was based on two pre-selected MRM transitions, relative ion intensity ratio and retention time. Even the two transitions worked well in matrices selected for validation studies, uncontrollable factors such as analyte concentrations in unknown samples, isobaric interferences, or signal suppression on the MRM transitions could lead to false positive or inconsistent identifications [39]. A full-scan EPI spectrum includes both molecular and fragment ions that can be used for confirmation, providing more confidence [24,40,41]. Identification or confirmation is potentially more definitive using one precursor ion and more than three product ions compared to only two product ions by MS/MS. Therefore, results for incurred dried fruit samples were further confirmed using a commercially available LC-MS mycotoxin library to confirm the identity of detected mycotoxins.

**Figure 3:**
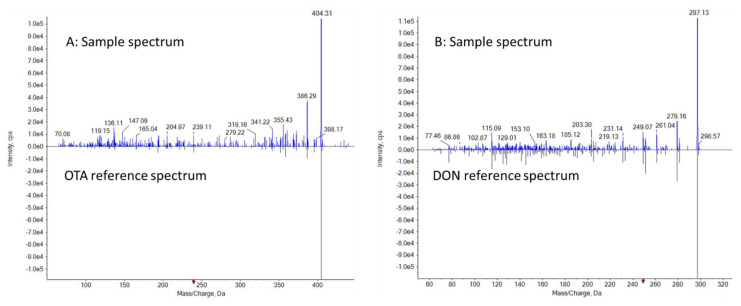
Mirror comparison of EPI spectra of OTA and DON collected from dried samples. (**A**): OTA Library search Fit = 84, Reverse Fit = 100, Purity = 84. (**B**): DON Library search Fit = 94, Reverse Fit = 95, Purity = 85.

Similar to NIST GC-MS library search [42], full scan product ion fragments of DON and OTA in the samples are compared and matched against the reference mass spectra in the mycotoxin library, and probable identities are confirmed based on forward and/or reverse-search mass spectrum matching results, Fit and ReverseFit scores, with 100 being the highest similarity [43]. The spectral matching values of detected OTA and DON are Fit = 84/100, Reverse Fit = 100/100 and Fit = 95/100, Reverse Fit = 94/100, respectively (Figure 3). Visually, one also could perform a library search by comparing the full scan product ion spectra from the incurred samples to the reference spectra in the LC-MS library. Figure 3 illustrates that a minimum of 3 characteristic fragments of OTA and DON could be matched within the spectra collected from the incurred samples with a retention time window ±45 s, providing additional confirmation for the presence of these mycotoxins.

## 4. Conclusions

In summary, we extended the application of stable isotope dilution LC-MS/MS to dried fruits. Our spike recovery data and the analysis of a small number of commercially available dried fruit products showed that the method identified and quantitated the majority of targeted mycotoxins in a time-efficient manner without using time consuming sample clean-up or matrix-matched calibration. For multi-mycotoxin analysis, method performance of individual mycotoxins may vary in some matrices, so it is necessary to prepare compound/matrix-specific troubleshooting tools to address issues related to sample homogeneity, matrix interferences, or confirmation of identity. As demonstrated in the study, if needed, the identity of detected mycotoxins could be confirmed using EPI-spectra. Furthermore, we developed troubleshooting procedures for the determination of DON and characterized sample homogeneity using laser diffraction particle size analysis. Overall, the study provided practical approaches focusing on analysis of multiple mycotoxins in dried fruits.

## Figures and Tables

**Table 1:** Particle size distribution of homogenized dried fruit samples and QC (NIST 1565 and CRM3310).

Dried Fruits and QC	Average Particle Size (RSD%), *n* = 10		
	Dv10 (µm)	Dv50 (µm)	Dv90 (µm)
Cranberry	23 (2)	165 (2)	556 (4)
Raisin	16 (8)	119 (8)	426 (13)
Plum	21 (2)	126 (3)	435 (8)
Fig	27 (3)	198 (3)	567 (5)
NIST1565 (measured)	31 (2)	225 (1)	550 (2)
NIST1565 (reference)	NA	NA	588 (3)
CRM3310 (measured)	41 (2)	71 (1)	116 (1)
CRM3310 (reference)	39 (5)	73 (4)	105 (6)

NA: not available.

**Table 2:** Average spike recoveries of target mycotoxins in dried fruit matrices.

Fortification Conc.	Matrix	Average Spike Recovery (RSD)%, *n* = 3											
		AFB1	AFB2	AFG1	AFG2	DON	FB1	FB2	FB3	OTA	T-2	HT-2	ZON
L-1 (1/10 ng/g)	Cranberry	89 (4)	105 (2)	101 (10)	99 (13)	ND	88 (21)	87 (5)	92 (2)	101 (13)	97 (6)	ND	107 (12)
	Fig	105 (2)	109 (7)	100 (4)	113 (1)	ND	255 (4)	124 (1)	ND	103 (5)	104 (8)	100 (19)	127 (12)
	Plum	114 (1)	112 (5)	104 (3)	145 (2)	ND	110 (6)	122 (1)	115 (4)	101 (4)	106 (9)	103 (9)	99 (5)
	Raisin	106 (2)	101 (2)	94 (3)	99 (3)	115 (12)	120 (7)	121 (3)	118 (4)	135 (1)	103 (4)	94 (11)	111 (7)
L-2 (10/100 ng/g)	Cranberry	87 (6)	98 (10)	104 (5)	113 (5)	98 (4)	88 (2)	92 (5)	90 (3)	95 (12)	98 (5)	89 (5)	100 (7)
	Fig	105 (3)	106 (3)	105 (4)	107 (1)	125 (6)	120 (2)	111 (2)	120 (1)	104 (1)	94 (6)	88 (1)	107 (2)
	Plum	112 (2)	112 (3)	105 (1)	124 (3)	ND	118 (4)	117 (2)	114 (2)	113 (4)	115 (1)	106 (5)	108 (4)
	Raisin	104 (2)	104 (5)	101 (3)	102 (4)	107 (6)	105 (6)	113 (2)	110 (1)	111 (5)	105 (3)	94 (9)	99 (1)
L-3 (100/1000 ng/g)	Cranberry	77 (5)	93 (5)	87 (4)	90 (6)	94 (5)	93 (6)	90 (8)	97 (3)	89 (4)	96 (8)	92 (9)	98 (6)
	Fig	104 (2)	107 (4)	104 (4)	110 (1)	107 (2)	110 (2)	110 (1)	111 (5)	107 (1)	101 (6)	92 (5)	104 (4)
	Plum	113 (1)	114 (4)	111 (5)	122 (8)	117 (5)	121 (3)	116 (1)	113 (4)	110 (1)	114 (5)	109 (11)	114 (1)
	Raisin	93 (3)	107 (2)	91 (14)	86 (8)	99 (5)	105 (3)	108 (2)	111 (6)	92 (13)	102 (5)	86 (14)	91 (8)

ND: not detected.

## Data Availability

Data available on request following Public Access to Results of FDA-Funded Scientific Research (https://www.fda.gov/science-research/about-science-research-fda/public-access-results-fda-funded-scientific-research, accessed on 17 February 2022).
